
JOURNAL INFORMATION
==============================
NLM Title Abbreviation: Schizophr Bull
Journal ID: Schizophr Bull
NLM Journal ID: 0236760
Journal ID: schbul

Schizophrenia Bulletin

ISSN: 0586-7614
EISSN: 1745-1701
Publisher: Oxford University Press

ARTICLE INFORMATION
==============================
PMCID: PMC5887537
DOI: 10.1093/schbul/sby018.893
Article ID: sby018.893
Article version: 1
Subjects: Abstracts, Poster Session III

S106. Submission Withdrawn

Print publication date: 2018 Apr
Electronic publication date: 2018 Apr 1
Volume: 44
Issue: Suppl 1
Issue title: Abstracts for the Sixth Biennial SIRS Conference
First page: S366
Last page: S366
Copyright: © Maryland Psychiatric Research Center 2018.
Copyright year: 2018
License: This is an Open Access article distributed under the terms of the Creative Commons Attribution License (http://creativecommons.org/licenses/by/4.0/), which permits unrestricted reuse, distribution, and reproduction in any medium, provided the original work is properly cited.
License URL: https://creativecommons.org/licenses/by/4.0/

Page count: 1

==============================
### S106. SUBMISSION WITHDRAWN

### S107. HEALTHCARE UTILIZATION AND COST IN SCHIZOPHRENIA AND BIPOLAR DISORDER: REAL-WORLD EVIDENCE FROM US CLAIMS DATABASES

Mallik Greene[1], Tingjian Yan[2], Eunice Chang[2], Ann Hartry*[,3], Jennifer Munday[4], Michael S. Broder[4]

*[1]Otsuka Pharmaceutical Development & Commercialization, Inc.; [2]Partnership for Health Analytic Research, LLC; [3]Lundbeck; [4]Phar, LLC*

**Background:** Schizophrenia (SCZ) and bipolar disorder (BD) are distinct psychiatric disorders, but patients may be diagnosed with both. The objective of this study was to explore healthcare resource utilization (HCRU) and cost in patients with claims-based diagnoses of SCZ, type 1 BD (BD-I), and both in a real-world setting.

**Methods:** This retrospective study used (1/1/12–6/30/16) Truven MarketScan® Commercial, Medicaid, and Medicare Supplemental databases. SCZ was defined as 1 inpatient or 2 outpatient claims for SCZ; BD-I was defined analogously. Three mutually exclusive groups were included: 1) SCZ alone: new episode with SCZ (e.g., met the claims-based diagnostic criteria for SCZ, but not for BD-I), 2) BD-I alone: new episode with BD-I (e.g., met the claims-based diagnostic criteria for BD-I, but not for SCZ), and 3) a diagnosis of both SCZ and BD-I: new episodes with both SCZ and BD-I (e.g., met the claims-based diagnostic criteria for both SCZ and BD-I). Descriptive statistics were reported; costs were adjusted to 2016 US$.

**Results:** Of the 63,725 patients in the final sample, 11.5% had SCZ alone, 80.8% had BD-I alone, and 7.7% had a diagnosis of both SCZ and BD. In the year following diagnosis, the group having a diagnosis of both SCZ and BD-I had the highest all-cause hospitalization rates (67.4% versus 39.5% in SCZ alone and 33.7% in BD-I alone) and the highest mean (SD) number of emergency room visits [3.44 (7.1] versus 1.39 (3.5) in SCZ alone and 1.29 (3.2) in BD-I alone]. All-cause total healthcare costs were highest in the group having a diagnosis of both SCZ and BD-I [mean (SD): $51,085 (62,759)], followed by the SCZ alone group [$34,204 (52,995)], and the BD-I alone group [$26,393 (48,294)].
